# Supplementary material for: Patterns of Intron Gain and Loss in Fungi
Source: PLoS Biol. 2004 Nov 30;2(12):e422. doi: 10.1371/journal.pbio.0020422 (PMC532390; doi:10.1371/journal.pbio.0020422)
Supplement: Table S1 — Also available at http://genes.mit.edu/NielsenEtAl/. (4.3 MB ZIP). [file pbio.0020422.st001.zip › NielsenEtAl/html/726.html]

AN3031.1.NCU03425.1.MG08923.1.FG05713.1


```
 CLUSTAL W (1.82) Multiple Sequence Alignments - Introns Inserted


Sequence 1: MG08923.1	543 aa
Sequence 2: FG05713.1	509 aa
Sequence 3: NCU03425.1	553 aa
Sequence 4: AN3031.1	517 aa
Alignment Length: 575 aa
Number Identitical Residues: 229 aa
Alignment Score (without introns) 12408


MG08923.1 	MENGAATN--GASEKSHSPSQTYLSTRGDDYG0LSFETVVLKGLAADGGLFLPEEVPAAT
NCU03425.1	MSNGVATNGTESQDQTHTPCQRYLSTRGEDGG0HSFEYVVLKGLAADGGLYIPQEVPVVP
FG05713.1 	----------MASDSTHTQSQVYLSTRGGDYG0---------------------------
AN3031.1  	--------------MSHTLSQKYLSTRGGSYG0FGFEEAVLKGLASDGGLFIPEEIPSLP
          	               :*: .* ****** . *  . .    .. ::...   ... .  .

MG08923.1 	E--W0QSWKDLPYTELAVKVLSLYISPAEVPTEDLRALVERSYSTFRSKEVVPLVKL--E
NCU03425.1	ESVW~QSWKDLSYTDLAYNIISLFVSPSEIPAEDLKDIINRSYATFRTPEVTPLVHL--Q
FG05713.1 	----~QSWKDLSYQELAFQIFSLYISRSEIPAEDLQGIINRSYSTFRAEEVVPLVQL--K
AN3031.1  	AGWE~TEWRDLSFEELAFNIMSLYISPSEIPSEDLRDIIKRSYSTFRHPERTPIVELDAK
          	 .    .*:**.: :** :::**::* :*:*:***: :::***:***  * .*:*.*.::

MG08923.1 	DNLHLLELFHGPSYSFKDC1ALQFLGNLFEYFL~TRKNKGKEGKD1RHHLTVVGATSGD0
NCU03425.1	DSIHLLELFHGPTFAFKDV~ALQFLGNLFEYFL~VRKNQGKEGRD~RYHLTVVGATSGD~
FG05713.1 	DNLHLLELFHGPSYSFKDC~ALQFLGNLFEYLL~TRKNEGKEGKD~RHHLTVVGATSGD0
AN3031.1  	QNLYLLELFHGPTFAFKDV~ALQFLGNLFEYFL0-----------~-----IVGATSGD~
          	:.::********:::***  ***********:*                  :******* 

MG08923.1 	TGSAAIYGLRNKKDVSVFILHPKGRVSPIQEAQMTTVLDQNVHNLAVTGTFDDCQ~DIVK
NCU03425.1	TGSAAIYGLRGKKDVSVFMLHPKGRVSPIQELQMTTVLDANVHNLAITGNFDHCQ0DILK
FG05713.1 	TGSAAIHGLRGKKDVSVTILHPKGRVSPIQELQMTTCTDANVHNLAVTGTFDDCQ0DIVK
AN3031.1  	TGSAAIYGVRGKKDVSIFMMFPTGKVSPIQQAQMTTVLDANVHNLTVEGSFDDCQ~DIVK
          	******:*:*.*****: ::.*.*:*****: ****  * *****:: *.**.** **:*

MG08923.1 	AMFNDPDSNATLKLGA~VNSINWSRILAQIVYYFHSYFSLARASPETFKVGDKVRFVTPT
NCU03425.1	SLFADPEINATHKLGA~VNSINWARILAQTVYYFYSYFSLARQSES-FNIGDKVRFVVPS
FG05713.1 	ALFGDPETNADLKLGA~VNSINFSRILAQIVYYFHSYFTLARKDPN-FKVGDKVRFVVPT
AN3031.1  	ALFADPDINSTHNLAA2IN---WSRVMAQVTYYFYSYFSLVKNTGS---DDSKVRFVVPS
          	::* **: *:  :*.* :*   ::*::** .***:***:*.:   .    ..*****.*:

MG08923.1 	GNFGNILAGYFAQKMGLPVDKLVV1ATN------E2FWKT-GRYEKKPASPEEAAGGLPQ
NCU03425.1	GNFGDILAGYFAFRMGLPVDKLVI~ATNENDSLDR~FWKT-GCYTKKPVSGSKAEGGLPE
FG05713.1 	GNFGDILAGYFATRMGLPVDKLVI~ATNENDILDR~FWKT-GKYEKQPAKGPEAEGGLEV
AN3031.1  	GNFGDILAGWFAKQMGLPTEKLVI~ATNENDILDR~FFRSNGQYTKNSGEGS--------
          	****:****:** :****.:***: ***...  .. *:::.* * *:. .          

MG08923.1 	DGVKAHEEGCKETLSPAMDILVSSNFERTLWFLAKE1FAATAGLNDEFNKKQAGQEVVAW
NCU03425.1	DGAKAHEDGVKETMSPAMDVLVSSNFERLLWYLAYE~FASSAGMDDEWNKKQAGQEVSSW
FG05713.1 	DGVKAHEDGVKETLSPAMDILVSSNFERLLWFLAYE~FAETVGMDVEFNRKQAGQEVATW
AN3031.1  	--------GVKETHSPAMDILVSSNFERLLWFLALR~TNTSSDSIEER-RKQASENVSQW
          	        * *** *****:******** **:** .    : .   *  :***.::*  *

MG08923.1 	YKSLKATGGFGPVHPEIMDNGRQVFESERVSDTQTLEMIAEMY----------------K
NCU03425.1	LTDLKVKDGFGPVYKDVLESARRNFESERVTDQETLDTIKSLY----------------Q
FG05713.1 	LKDLKNKGGFGPVYVDVLNSARKTFESERVSDPQTLETIKDTY----------------Q
AN3031.1  	LSQLKTEGGF-TVPPAVIEAAKTEFESERVSNDETIAQIRSTYNSSFPSNLTPGSATSSK
          	 ..**  .** .*   ::: .:  ******:: :*:  * . *.:: .:. :..::::::

MG08923.1 	ATKYVLDPHSAVGVAGAKRSMSRASN-VPHIALSTAHPAKFSGAVELALKDQKEFDFTKQ
NCU03425.1	EVGYVLDPHTAVGVEAARRSVKRAGADIPHISLSTAHPAKFSGAVNLALQDQPEFDFEAK
FG05713.1 	KLGYILDPHSSVGITAALRSAERAEAKIPHISLSTAHPAKFAGAVELALKDEKDFNFNEK
AN3031.1  	TGGYILDPHSAVGVAASLRSIQRNPG-TKHISLSTAHPAKFASAVDLALRAEDGYDFT-E
          	   *:****::**: .: ** .*      **:*********:.**:***: :  ::*  :

MG08923.1 	VLPEDFVGLAEKEKRVTEVAA--NWQEVREIVKKQVEEDLKAESSA
NCU03425.1	VLPQEFVGLDQKEKRVQDVAN--DWTAVREVIREQVEEELKALDSA
FG05713.1 	VLPEEFVGLEKLPKRVSDISN--DWKAVREQVKKQVAEELGGQ---
AN3031.1  	VLPQEFIGLEQRESRVTPVRAGAGWEGVREIVKAEVEQELEGKR--
          	***::*:** :  .**  :  .:.*  *** :: :* ::* .
```
